# The effects of the Omagh bomb on adolescent mental health: a school-based study

**DOI:** 10.1186/s12888-015-0398-9

**Published:** 2015-02-06

**Authors:** Michael Duffy, Maura McDermott, Andrew Percy, Anke Ehlers, David M Clark, Michael Fitzgerald, John Moriarty

**Affiliations:** Queens University, Belfast, Northern Ireland UK; Western Health and Social Services Trust, Omagh, Northern Ireland UK; University of Oxford, Oxford, UK; Trinity College Dublin, Dublin, Ireland

**Keywords:** PTSD, Bombing, Cognitive models, Adolescents, School survey

## Abstract

**Background:**

The main objective of this study was to assess psychiatric morbidity among adolescents following the Omagh car bombing in Northern Ireland in 1998.

**Methods:**

Data was collected within schools from adolescents aged between 14 and 18 years via a self-completion booklet comprised of established predictors of PTSD; type of exposure, initial emotional response, long-term adverse physical problems, predictors derived from Ehlers and Clark’s (2000) cognitive model, a PTSD symptoms measure (PDS) and the General Health Questionnaire (GHQ).

**Results:**

Those with more direct physical exposure were significantly more likely to meet caseness on the GHQ and the PDS. The combined pre and peri trauma risk factors highlighted in previous meta-analyses accounted for 20% of the variance in PDS scores but the amount of variance accounted for increased to 56% when the variables highlighted in Ehlers and Clark’s cognitive model for PTSD were added.

**Conclusions:**

High rates of chronic PTSD were observed in adolescents exposed to the bombing. Whilst increased exposure was associated with increased psychiatric morbidity, the best predictors of PTSD were specific aspects of the trauma (‘seeing someone you think is dying’), what you are thinking during the event (‘think you are going to die’) and the cognitive mechanisms employed after the trauma. As these variables are in principle amenable to treatment the results have implications for teams planning treatment interventions after future traumas.

## Background

A considerable amount of research has been published on the psychological effects of traumatic events on children, adolescents and adults. Although a range of mental health problems develop after trauma, the most common disorder reported is post traumatic stress disorder (PTSD) [1] which is associated with intrusive memories of the event, hyperarousal symptoms and avoidance of reminders [2].

### The Omagh Bombing

On 15 August 1998, the largest single atrocity of the Northern Ireland conflict took place in Omagh, a market town with a population of 26,000, when a car bomb exploded in the town centre. Thirty-one people, including two unborn children (twins) were killed and 15 of the deceased were aged 17 years or under. Almost four hundred people were injured of which 135 were hospitalised. Many children and young people sustained injuries resulting in the loss of limbs, loss of soft tissue, scarring and disfigurement. Many more were exposed to the scenes of death, injury and destruction after the explosion. The local Health and Social Care Trust decided to assess the psychological effects of the bombing through three studies; an adult community study [3], a school based children study [4] and a school based adolescent study. This paper presents the findings of the adolescent study.

The primary aim of this study was to assess the extent of psychiatric morbidity among adolescents (aged 14 to 18 years) after the bombing. Secondly, we were interested to discover how exposure type relates to PTSD and general emotional distress. Thirdly we investigate which individual and trauma characteristics predict chronic PTSD symptoms and we consider peri and post trauma factors recommended for further investigation by previous reviews [5] that may help inform therapeutic responses to other traumatic events. In relation to the first aim, although children and adolescents can develop PTSD in response to a diverse range of stressors [6] the literature reports a wide variation in PTSD rates [7] even after similar types of traumas [8]. In natural disasters PTSD rates between 1- 95% have been reported [9] whereas much higher rates between 25-70% have been reported in warfare studies [10]. This study explores PTSD in adolescents after a conflict-related human inflicted trauma.

In terms of the second aim, a number of adolescent studies have reported increased levels of exposure as a significant risk factor for PTSD [11-13] and other psychological problems [14]. However the concept of trauma exposure and relationship between exposure and psychopathology needs further investigation.

In a meta-analysis of PTSD risk factors in children and adolescents Trickey and colleagues [5] pose a number of questions about definitions of trauma exposure and trauma severity and ask how these can be adequately differentiated. We were interested in exploring whether specific aspects of a trauma (such as seeing some-one die) are more important in determining the severity of the negative psychological effects of the trauma. In our study we also consider the concept of “near miss” which has to the best of our knowledge had not been researched prior to the Omagh bombing studies. We were interested in the possibility that adolescents may be traumatized by knowing they might have been harmed and perhaps later engaging in rumination which is increasingly recognised as an important maintenance factor in PTSD [15,16].

In respect of the third aim, the factors that may be associated with PTSD and general psychological distress can be categorised as demographic and pre trauma characteristics such as age and gender; type of exposure; peri trauma reactions; post trauma psychological reactions and environmental or social factors. In considering pre trauma factors, younger age has been identified as a significant but weak risk factor in adults [17] and children [11,18]. However, reviews have reported the lack of consistency of age as a predictor of whether an adolescent will develop PTSD [5]. Similarly, gender has also been reported as a significant but weak risk predictor in adults [17] and children and adolescents [11,18]. The association between gender and PTSD in children and adolescents increases with age [5] which may be partly explained by the tendency for increased rumination in females [19] a maintenance factor also included in Ehlers and Clark’s cognitive model for PTSD [20]. In respect of peri and post traumatic risk factors it is recognised that adolescent responses to trauma differ from children and more closely resemble adults due to developed cognitive abilities and increased capacity for encoding information and appraisals of the event [21].

A review of predictors of PTSD in adults by Ozer and colleagues [22] found that peri traumatic emotions, peri traumatic dissociation and perceived life threat were amongst the highest reported risk factors albeit with small effect sizes. In children and adolescents perceived life threat was also found to be associated with the onset of PTSD [23] and a consistent predictor after accidental traumas [11]. In Trickey and colleague’s meta-analysis [5] perceived life threat was reported as a risk factor with a large effect size. Although Trickey and colleagues review [5] found that negative appraisals and emotions during a trauma contribute to risk of PTSD few studies were found which researched these factors. This study responds to a recommendation in their review for further research in relation to peri-traumatic emotions and cognitions.

In terms of post trauma risk factors Brewin and colleague’s review of PTSD in adults [17] reported lack of social support as the highest risk factor for PTSD (effect size 0.4) and in Ozer’s adult review [22] low perceived social support was reported as the second highest risk factor (effect size 0.28). In Trickey and colleagues [5] review of post-trauma factors for children and adolescents, social support was identified as important and poor family functioning was a stronger risk factor for PTSD than poor parental mental health. However the lack of research was again noted with only four studies located that adequately addressed the concept of social support.

In both the adult reviews [17,22] demographic and pre-traumatic factors such as age, gender, family psychiatric history, prior psychiatric history and prior trauma experiences were significantly but weakly associated with risk of PTSD with small effect sizes whilst stronger associations were reported for peri and post-traumatic factors although only with small to medium effect sizes. In Trickey and colleague’s review [5] of risk factors in children and adolescents a similar pattern emerges with demographic and pre trauma factors reported with only small to medium effects, peri traumatic emotions and cognitions with large effects and post traumatic factors such as co-morbidity, distraction and thought suppression reported as major risk factors.

Many of the psychological factors that have been found to be important predictors of PTSD have been specified in Ehlers and Clark’s cognitive model [20]. The model proposes that PTSD is maintained by three inter-related mechanisms; negative beliefs about the trauma and its sequealae; problematic aspects of the trauma memory and counter-productive strategies for dealing with the symptoms. In two adult studies Ehring, Ehlers and Glucksman [24,25] compared a range of factors specified in Ehlers and Clark’s [20] cognitive model of PTSD with the factors identified in previous research [17,22]. The cognitive factors were substantially more powerful in predicting PTSD. Trickey and colleagues [5] suggest a cognitive model may offer a framework for investigating the mechanisms by which PTSD is triggered and maintained in adolescents. The present study investigates whether the psychological factors proposed in Ehlers and Clark’s cognitive model may be helpful in predicting chronic PTSD in adolescents following a bombing.

## Method

Full ethical approval for the survey was granted by the Sperrin Lakeland Health & Social Care Trust (SLT) which was the relevant ethical and institutional body at the time (1999). The Trust secured the agreement and assistance of the Western Education & Library Board, the main regulatory body for schools in the Omagh area and school principals to survey children in the classrooms. The SLT stated that the findings from the Omagh study should be disseminated and published for potential benefits to other communities responding to such traumatic events. A passive consent procedure was used to obtain parental consent, that is to say all parents were informed of the study and asked to reply, via prepaid envelope, if they wished their young person to be excluded from the study. Parents who consented to participation did not have to reply. The parents of bereaved young people and those who were hospitalised or already receiving therapy were contacted directly by members of the Omagh Trauma and Recovery Team about the study. Briefing meetings were held with all school principals, senior teaching staff and teachers with responsibility for pastoral care in the schools.

Data was collected 15 months after the car bomb and involved close collaboration between local education and health authorities. All adolescents aged between 14 and 18 years who were registered within mainstream secondary level schools within the Omagh area were eligible for inclusion. All post primary schools participated in the study providing a response rate of 83% of all adolescents in secondary level schools in the Omagh area. Demographic information on the sample is shown in Table 1 and indicates a typical distribution of age and gender, with slightly more girls remaining in school during adolescence. Data was collected via a self-completion booklet, completed in groups within schools. All fieldwork was undertaken and supervised by a professional survey organization and local child and adolescent mental health professionals were available in each school at the time of completion for any young person requiring support but none were required to intervene. Contact details of the Trauma and Recovery Team and other support services were made available to participants, parents and schools to access assistance after the study.

**Table 1:** **Frequency and descriptives for predictor variables**

	**Frequency**	**Mean (SD)**
Female	1,162 (52.32%)	
Male	1,059 (47.68%)	
Age		15.85 (1.24)
Prior psychological help	87 (3.92%)	
*Household structure*		
Both biological parents	1,943 (88%)	
Single parent household	218 (9.87%)	
Reconstituted household	47 (2.13%)	
*Exposure to trauma*		
Present when explosion happened	33 (1.49%)	
Person hurt	42 (1.9%)	
Person thought he/she was going to die	35 (1.5%)	
Witness shortly after explosion	445 (20.0%)	
Person saw dead others	153 (6.9%)	
Person saw others going to die	213 (9.6%)	
Person saw other people hurt	297 (13.4%)	
Someone close to person died	527 (23.7%)	
Someone close to person was hurt	1153 (51.9%)	
*Derived exposure categories*		
Present when explosion happened	33 (1.49%)	
Witness after explosion	295 (13.28%)	
Loss	370 (16.66%)	
Near miss	121 (5.45%)	
No exposure	1402 (63.12%)	
*Cognitive predictors*		
PTCI factor A		30.40 (15.40)
PTCI factor B		12.38 (4.05)
Thought/Emotional suppression		8.07 (3.47)
Rumination		8.47 (2.89)
“Nowness” of memory		1.45 (1.03)
Muddled memory		1.71 (1.12)
*Response factors*		
Felt more part of community	925 (41.65%)	
Post psychological help	70 (3.15%)	

### Measures

The questionnaire contained basic demographic questions and 10 items relating to the degree of exposure to the bombing. Respondents were classified as belonging to one of five mutually exclusive levels of exposure. “Present” means the respondent was in town when the explosion happened and answered yes to at least one of the four exposure questions (injured, saw people dead, or about to die, saw people hurt). “Witness” means the respondent was *not* in town at the time of the explosion but arrived afterwards and answered yes to at least one of the exposure questions. “Loss” means the respondent was *not* in town at the time of explosion or a witness but experienced loss of someone to whom they were close. “Near miss” means the respondent was in town shortly before the explosion but was not hurt, did not witness death or injury and did not experience loss. “No exposure” means the respondent was not in town that day, was not a witness, and did not experience loss.

PTSD symptoms were assessed by the Posttraumatic Diagnosis Scale [26] a validated and widely used self-report measure of PTSD severity and probable PTSD caseness. The instructions explicitly mentioned the Omagh bomb. Respondents with a PDS score of 20 or more were considered probable PTSD cases [26]. General psychiatric problems were assessed by the 12-item General Health Questionnaire [27] a well-validated and widely used self-report measure for assessing common psychiatric symptomatology and probable caseness in primary care. GHQ-12 items were scored in the conventional manner (0,0,1,1) with an overall score of 4 or more indicating probable casesness [27]. The caseness cut-offs for both the PDS and the GHQ were chosen so they were among the most conservative that have been used in previous studies. Post-trauma beliefs were assessed by a shortened version of the Post-traumatic Cognitions Inventory (PTCI) [28] which has been shown to have good reliability and convergent validity and to discriminate between traumatized people with and without PTSD. A principal components factor analysis with varimax rotation identified two main PTCI factors in the survey population. Factor A, represented by 14 items, comprises negative beliefs about oneself and the symptoms of PTSD (e.g. “My reactions since the bombing mean I am going crazy”, “There is something wrong with me as a person”, “I can’t rely on myself”). Factor B, represented by 3 items, comprises beliefs about the world being an unsafe place. (e.g. “You never know who will harm you”, “I have to be on guard all the time”). Qualities of trauma memories were assessed by questions from previous research [29,30] and measured the disorganisation (“muddled, unclear”) and perceived nowness (“seem to be happening now instead of being something from the past”). Responses to memories were assessed with shortened versions of the Response to Intrusions Questionnaire [31,32] assessing rumination (e.g., “I dwell on what life would have been like if the bombing had not happened”) and suppression of thoughts and emotions (e.g., “I try hard to push them out of my mind”).

### Statistical analysis

In order to determine whether increasing exposure to the events connected with the bombing had a greater effect on PTSD symptoms than on general psychiatric symptoms, PDS and GHQ total scores were each converted to standard scores (mean = 0, SD = 1). For the probable casesness data Odds Ratios with respect to no exposure were compared for each exposure category. As a proportion of respondents failed to complete some questionnaire items probable caseness analysis was restricted to those with valid scores on the PDS measure (N = 2095) and GHQ measure (N = 2155) (Table 2). In participants with exposure to the bomb regression analyses tested the association between potential predictors and PDS and GHQ total scores, controlling for the main pre trauma factors, age, gender and family structure or alternative family type (Table 3). In order to consider the effects of pre, peri and post trauma factors a series of hierarchical regression models were constructed on conceptually related groups to examine the main predictors of reported PDS and GHQ scores (Table 4). Pre trauma factors including basic demographics (gender and age), and prior psychological problems were entered into an initial baseline model (model 1); dummy variables representing exposure experiences (peri trauma factors - level of exposure and perceived life threat) were entered in model 2; post trauma factors linked to Ehlers and Clark’s cognitive model for PTSD (PTCI factors A and B, memory factors, rumination, thought/emotion suppression) were added in model 3; and the environmental post trauma factors, family type and structure, perceived community cohesion and social support, were added in model 4. For most questionnaire items a proportion of respondents failed to complete the item. Among those who reported direct exposure (either present or as a witness after the explosion) the missing data rates were generally lower. Models 1 and 2 used all participants with a valid score for PDS (N = 2095). Dummy variables representing missing information relating to each exposure experience were included in all regression analyses and were not significantly associated with the PDS outcome. For models 3 and 4, N was restricted to those with valid scores on post-trauma cognition scales. An additional sensitivity analysis tested whether results in model 2 were altered after dropping those with no valid score on the cognitive variables. No difference in results was detected, suggesting that having missing information was largely orthogonal to key outcomes. The only exception to this was a significant effect for having a missing value on the *nowness of memory* item: participants who did not answer this question had higher PDS scores than those who answered “No”.

**Table 2:** **Caseness rates according to the Posttraumatic Diagnosis Scale (PDS > 20) and the General Health Questionnaire (GHQ > 3)**

	**PDS Cases (percentage of 2095 valid responses)**				**GHQ Cases (percentage of 2155 valid responses)**			
	**N**	**%**	**OR**	**CI**	**N**	**%**	**OR**	**CI**
No exposure	22	1.7	-	-	181	13.4		
Near miss	6	5.3	3.86**	1.50-9.95	24	20.5	1.75*	1.06-2.89
Loss	17	4.8	2.79**	1.45-5.38	68	18.5	1.40*	1.02-1.92
Witness	48	17.3	13.75**	7.86-24.08	93	32.5	3.20**	2.31-4.44
Present	10	32.3	26.55**	8.32-84.63	13	39.4	4.07*	1.62-10.24
Total	103	4.9			379	17.6		

Note: OR = odds ratio for probable caseness compared to the no exposure group. CI = the 95% confidence interval for the OR estimate. *p < 0.05; ** p < 0.01. In the calculation of the OR, the logistic model controlled for gender, age and family structure and having a missing value on any of the items used in the construction of the exposure scale (parameter estimates for these covariates are not shown). N is restricted to those with valid responses on the PDS and the GHQ.

**Table 3:** **Correlations with symptom scores on the PDS and GHQ in young people with direct exposure of the explosion at the time or shortly afterwards**

	**PDS (N = 2095)**		**GHQ (N = 2155)**	
	**Bivariate correlations**	**OLS** ^**a**^	**Bivariate correlations**	**OLS** ^**a**^
In town at time of explosion	0.35	2.02** (0.62)	0.19	0.15 (0.27)
In town after explosion	0.26	0.47 (0.49)	0.15	0.06 (0.21)
Was hurt	0.33	4.31** (1.63)	0.21	2.39** (0.71)
Left with injury or scar	0.29	−1.1 (2.04)	0.15	−0.51 (0.89)
Saw doctor afterwards	0.38	7.25** (1.38)	0.23	1.68** (0.61)
Attended hospital	0.32	0.95 (1.77)	0.15	−1.23 (0.78)
Saw people who were going to die	0.35	2.23** (0.8)	0.19	0.29 (0.35)
Saw people dead	0.33	1.41 (0.81)	0.19	0.41 (0.36)
Person close to them hurt	0.23	1.55** (0.41)	0.20	0.82** (0.18)
Person close to them died	0.23	2.37** (0.36)	0.14	0.36* (0.16)
Other acquaintance hurt	−0.14	0.45 (0.42)	−0.12	0.27 (0.18)
Other acquaintance died	−0.03	0.54 (0.31)	−0.03	0.00 (0.13)
Life was under threat	0.27	3.4** (1.17)	0.16	1.39** (0.51)
Saw others hurt	0.33	0.27 (0.7)	0.19	0.32 (0.31)

Note: *p < 0.05; **p < 0.01. Sample is of all participants with valid scores on both PDS and GHQ. The model controlled for gender, age and family structure and having a missing value on any of the exposure items (parameter estimates for these covariates are not shown).

a. Ordinary Least Squares.

**Table 4:** **Predictors of PTSD symptoms at 15 months after the explosion among young people who were in town at the time of, shortly after or left just before the explosion**

	**Model 1**	**Model 2**	**Model 3**	**Model 4**
	**(N = 2095)**	**(N = 2095)**	**(N = 1856)**	**(N = 1848)**
R-squared	0.19	0.22	0.56	0.59
Model fit (RMSE)^a^	0.96	0.89	0.65	0.64
Gender (Female)	0.41** (0.04)	0.34** (0.04)	0.01 (0.03)	0.01 (0.03)
Age	−0.06** (0.02)	−0.07** (0.02)	−0.01 (0.01)	−0.02 (0.01)
Prior psychological help	0.65** (0.11)	0.49** (0.1)	0.21** (0.08)	0.13 (0.08)
Exposure				
*Near miss*	-	0.19* (0.09)	0.04 (0.07)	0.04 (0.07)
*Loss*	-	0.30** (0.05)	0.11* (0.04)	0.10* (0.04)
*Witness*	-	0.84** (0.06)	0.38** (0.05)	0.30 (0.05)
*Present*	-	1.16** (0.17)	0.58** (0.13)	0.37** (0.13)
Perceived life threat	-	1.26** (0.16)	0.56 (0.14)	0.34* (0.14)
Cognitive predictors				
*PTCI factor A*	-	-	0.03** (0)	0.03** (0)
*PTCI factor B*	-	-	0.01 (0)	0.01 (0)
*Thought/Emotional suppression*	-	-	0.04** (0.01)	0.03 (0.01)
*Rumination*	-	-	0.04** (0.01)	0.04** (0.01)
*“Nowness” of memory*	-	-	0.16** (0.02)	0.16** (0.02)
*Muddled memory*	-	-	0.02 (0.02)	0.01 (0.02)
Social support & family structure				
*Single parent*	-	-	-	0.00 (0.05)
*Reconstituted*	-	-	-	0.12 (0.11)
*Felt more part of community*	-	-	-	0.02 (0.03)
*Post psychological help*	-	-	-	0.79** (0.09)

Note: *p < 0.05; **p < 0.01. In the calculation of the regression model the outcome variable was standardized (z score) and the model controlled for having a missing value on any of the items used in the construction of the exposure scale (parameter estimates for these covariates are not shown).

^a^Root Mean Square Error.

## Results

### Type of exposure and caseness

**Figure 1:**
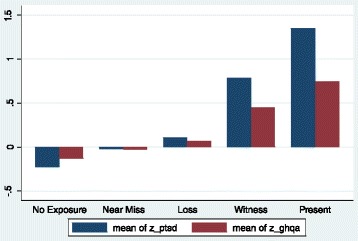
**PDS and GHQ standard scores for types of exposure.**

Distributions of the derived exposure categories, the experiences underlying these categories and mean scores for the putative cognitive mechanisms are provided in Table 1. Figure 1 shows the PDS and GHQ standardized symptom scores for each type of exposure. A pattern of increases on both measures with increased levels of exposure is evident but in the two exposure groups, those present at the time of the explosion and those present after the explosion, there is a proportionally larger increase in PTSD symptoms relative to general psychiatric distress. The odds ratios for clinical caseness on the PDS and GHQ are shown in Table 2. While the numbers of adolescents reporting symptoms of probable caseness are relatively small (n = 103 PDS caseness; n = 379 GHQ caseness), those young people exposed to the bombing are significantly more likely to be classified above the case threshold on both the PDS and GHQ than those young people who reported no exposure to the bombing. Those young people who reported a more direct physical exposure to the bombing were significantly more likely to score above the caseness threshold. Although this effect was evident for both PDS and GHQ caseness, it was much more marked for the former. Among young people who were in present at the time of the explosion the OR for probable PDS caseness is 26.55 compared to 3.20 for probable GHQ casesness. Similarly among witnesses after the explosion, the OR for probable PDS caseness is 13.75, compared to 3.97 for probable GHQ caseness. The absolute rates of probable PDS caseness are 32.3% for young people who were in present at the time and 17.3% for witnesses afterwards. It is worth noting that the confidence intervals associated with the odds ratios of each exposure category tend to overlap. This suggest that exposure category, in itself, is not a particular strong discriminator of clinical caseness and that other processes may be involved in the determining the severity of the clinical outcome experienced by adolescents.

### Predictors of PTSD amongst those exposed to the bombing

The correlations in Table 3 report the associations between the exposure variables and the outcome measures the PDS and GHQ when controlled for the pre trauma factors of gender, age and family structure. The significant predictors are; being in town at the time of the explosion, being hurt, seeing people who you thought were going to die, perceived life threat, having someone close who died or was injured, or seeing a doctor after the event. Interestingly, being in town afterwards was not significantly associated with either outcome measure but this may simply mean that some of the respondents in town afterwards were not exposed to post trauma distressing scenes. Therefore being in town before or after was combined with at least one positive response to the more detailed exposure questions (seeing someone die, injured, about to die, being hurt) to more accurately categorise the respondents into exposure type sub groups for the next set of regression analyses reported in Table 4.

In Table 4, regression models 1 and 2, being female, being younger and having received prior psychological treatment each predicted higher PDS scores. In Model 2 elevated PDS scores are found for individuals in all four of the exposure categories compared to the zero exposure reference category. The perception that one’s life was under threat is also strongly associated with the PDS. Post hoc analysis of the exposure categories showed stronger effect on the PDS of having witnessed either the explosion or the aftermath over having lost someone close to them or experienced a “near miss”. However, in all models, there was no significant difference in the effect on the PDS between having been present during the explosion and having witnessed the aftermath. Nor was there any significantly greater effect of having experienced loss over having experienced a near miss. This suggests some face validity of this categorization of exposure and some discriminant validity in distinguishing traumatic grief from post trauma stress responses.

When the cognitive variables were included in the analysis (model 2 and 3) associations between the PDS and both being female and being younger fall to zero^a^. Exposure, in contrast, remained a significant predictor of PDS scores even after the introduction of the cognitive variables, although the near miss category was no longer associated with a significant increase in PDS scores above the no exposure group. This provides some evidence that the elevated PDS scores among females, younger adolescents and those who narrowly avoided direct exposure are attributable to how those groups of young people cognitively process events later on. Amongst the cognitive variables, the main predictors were the PTCI factor A, rumination, thought and emotional suppression and the sense of remembering aspects of the trauma as if happening in the present moment.

When the non-cognitive post trauma variables were added to the analysis only “having received help” for difficulties experienced as a result of the bomb was found to be significant whereas family structure or alternative family type was not found to be a significant predictor of PDS scores nor was a sense of community cohesion. The combined pre and peri trauma risk factors highlighted in previous meta-analyses, age, gender, level exposure, exposure characteristics (see Table 3) and perceived life threat at the time, account for 20% of the variance (adjusted R-squared) in PDS scores. However when the variables highlighted in Ehlers and Clark’s cognitive model of PTSD [21] are added the amount of variance accounted for increases to 56%.

## Discussion

The primary aim of the study was to assess psychiatric morbidity after the explosion in adolescents. Although less than two per cent of the sample was present at the time of the explosion twenty per cent were witnesses of horrific scenes after the explosion, including those who saw the remains of the deceased or saw others injured. The higher proportion of females present is probably explained by the tendency of adolescent females to socialise with friends in town on Saturday afternoons. Almost a quarter of the sample reported loss of someone close. This is understandable given the high proportion of children and young people who died in the explosion and the social inter-connections amongst adolescents in a small market town. The results presented in Table 2 suggest numbers meeting probable caseness for PTSD and general psychiatric problems are relatively small. The results also suggest an underlying level of mental health problems within the adolescent population with 1.7% of the group that was not exposed to the Omagh bombing meeting probable caseness for PTSD on the PDS and 13.4% meeting caseness on the GHQ. Eighty seven (3.92%) of the respondents reported receiving professional help for psychological problems before the bomb.

The second aim of the study was to investigate negative psychological responses as a function of different levels of exposure to the bombing. The results suggest that level or type of exposure to potentially traumatic events predicts general emotional distress but more specifically predicts probable PTSD in young people. The difference can be observed in the chart (Figure 1) and is consistent with the findings in the Omagh bombing adult [3] and children studies [4] and other studies of adolescent PTSD after traumatic events [11-13]. An interesting question posed by Trickey and colleagues [5] is how to distinguish between different levels of trauma severity. Our finding that “seeing people you thought were dying” was a significant predictor of PDS scores but “seeing people you thought were dead” was not significant suggests that there may be idiosyncratic features of a trauma that are more relevant to predicting risk. Interestingly, in the study of younger children after the Omagh bomb [4] the only exposure factor from a similar list that was found to be significant was “seeing people injured”.

As indicated earlier, we were interested in considering the concept of “near miss” given the variation in many published studies of trauma responses to different degrees of exposure and the increasing recognition of factors such as rumination in PTSD [15,16]. The positive “near miss” association with PDS scores in this study is different to the findings in the Omagh adult study [3] which found no significant difference in PTSD symptoms between the no exposure and near miss groups. This association in adolescents may be explained by cognitive factors such as rumination (e.g. “what if I had not left at that time”) which has already been reported as an important onset and maintenance factor in adolescent PTSD and particularly in females [19,33]. The prolonged media coverage containing graphic video coverage of the immediate aftermath of the bomb may have been a factor in encouraging rumination and the increased risk of PTSD in the near miss group [34,35].

The final aim of the study was to identify specific predictors of chronic PTSD amongst those adolescents directly exposed to the bombing. In terms of pre trauma factors gender has often been reported as a risk factor for PTSD particularly in older children [5,11,18] but as already discussed, adolescent females may be at greater risk of emotional distress due to a tendency toward rumination [33,36]. In this study rumination had a moderating effect on gender as a risk factor. While in-depth discussion of such interactions is beyond the scope of this paper, this finding gives tentative evidence that between-gender differences in rumination may account for much of the between-gender differences in PTSD. It is also worth noting that there were significantly more females exposed to the bomb, either present at the time of the explosion or as witnesses afterwards (χ^2^ = 58.48, *df* = 5, p < 0.001). Collectively the factors explained by Ehlers and Clark’s cognitive model mediated the effect of gender and also younger age entirely.

Although being hurt at the time and attending a family doctor after the event were associated with higher PDS scores, long term physical injury or scars was not a significant factor (Table 3). This finding is different from some studies in conflict related PTSD in adolescents [11,37] and may be due to the small numbers who reported “long term injury or scars” in this sample (n = 30)]. In Cox and colleagues meta–analysis [11] physical injury was reported to be significant but only a weak predictor of PTSD in children and adolescents.

The only social support variable that was significantly associated with PDS scores after controlling for the cognitive factors was “receipt of help for problems experienced since the bomb” from a list of sources including family doctor, counsellor, therapist psychologist, teacher, social worker and psychiatrist. Social support has been identified in reviews elsewhere as a risk factor with a large effect in both adults [17] and children and adolescents [5] but requires further investigation to delineate the specific elements of this factor. In this study neither a sense of community cohesion nor family structure (living with one or both parents or one parent and partner number of siblings) type (living with family, in foster care or in residential care) were not found to be significant. Our findings are in line with Ma and colleague’s study that found social support to lessen the impact of an earthquake on adolescent mental health by affecting post-trauma negative cognitions [38].

The cognitive variables derived from Ehlers and Clark’s [20] cognitive model of PTSD account for most of the variability in the PDS outcomes suggesting the impact of exposure is mediated via post trauma cognitive processes. This findings is consistent with the Omagh bombing adult study [3] and other studies that highlight the importance of post trauma perceptions in adolescents [39], the harmful effects of thinking styles such as thought suppression [16] negative appraisals and negative coping styles [37,40]. These findings are in line with previous research in adults that focused on motor vehicle accidents, assault or emergency workers [24,32,41] and in children and young people [7,42]. A cognitive therapy programme that specifically focused on the psychological variables identified in this study and the Omagh adult study [3] was provided for adults and older adolescents (17 plus) after the Omagh bombing and was associated with large reductions in PTSD [43]. Similar encouraging results were reported in a randomized controlled trial [44] with a more chronic PTSD group linked to the Northern Ireland conflict and more recently when cognitive therapy for PTSD has been made available to children and adolescents [45].

Trickey and colleagues [5] reported that the relationship between pre-trauma life events and PTSD is significant but modest in comparison to certain peri-trauma and posttrauma factors. This study supports the suggestion that PTSD in adolescents is primarily associated with their reaction to the specific event, rather than previous characteristics of the young person. The findings in our study suggest that exposure alone is not a precise predictor of risk rather it is the aspects of a trauma that the young person is exposed to (seeing someone you think is dying); what you are thinking during the event (think you are going to die); and the cognitive mechanisms employed thereafter, particularly if a young person develops negative beliefs about oneself or the PTSD symptoms, ruminates, and the memory retains a sense of the trauma still being in the present.

These findings may have important clinical value for therapists and planners responding to large scale traumatic incidents and have been made available to therapists responding to other large scale disasters and tragedies such as the 2011 mass shootings in Norway. Teams planning the treatment response to other bombings, such as the Boston Marathon bombs, may also find the results useful.

Finally, school based screening and assessment instruments have been used successfully elsewhere [46,47] and we found this mechanism useful for early identification of adolescents who may require therapeutic intervention.

### Strengths and limitations

The main strength of the study is that the data were drawn from a large scale population survey of adolescents attending school in the town where the bomb was located. This recruitment strategy achieved a large representative sample of school-going adolescents, allowing for inquiry into the outcomes of subgroups differently affected by the Omagh bombing. The non-selectivity of the sample contributes to the reliability of the results presented above. In addition, data was collected in a neutral environment (the classroom) employing widely used and well validated measures.

However, our data were gathered 15 months after the bomb but did not capture any traumas or significant life events that may have been experienced in the intervening period. Self-report questionnaires were used in the screening and we accept these are only an indicator of probable psychiatric disorders and do not provide a complete diagnosis. We were unable to collect multi-informant data from parents or teachers which would have provided confirmatory data to identify morbidity amongst the sample. As all data were collected within a cross-sectional survey, the particular relationship between *troublesome trauma-related cognitions* and *post-traumatic stress* is difficult to disentangle, as either may have occurred first.

### Endnote

^a^Further iterative analysis revealed that after adjusting for levels of rumination alone, the association between gender and PTSD caseness falls from 0.4 (p < 0.1) to 0.2 (p = 0.09).
